# Supplementary material for: Content-rich biological network constructed by mining PubMed abstracts
Source: BMC Bioinformatics. 2004 Oct 8;5:147. doi: 10.1186/1471-2105-5-147 (PMC528731; doi:10.1186/1471-2105-5-147)
Supplement: Additional File 2 — The original results of the above study (non-essential files are deleted to keep the file size under the limit set by BMC bioinformatics). [file 1471-2105-5-147-S2.bz2 › chilibotAdditionalFile2/dip05/431ID10910894E10361/html/CCL16_CCR8.html]

 


 **CCL16** and **CCR8** 
  
Found 7 abstracts in PubMed, retrieved 05.  
 

 What does Google say? 
 PDF only 
| .edu only 

---

**Interactive relationship** (e.g. stimulation, inhibition, etc)

**Neutral relationship**- LEC  [ **CCL16** ]  has been shown to chemoattract monocytes, lymphocytes, and polymorphonuclear leukocytes PMNs by its binding to CCR1 and  **CCR8**  chemokine receptors.  Ref: 12843794 J Immunother< MedlineTA>J Immunother,

**Non-interactive relationship** (e.g. studied together, co-existance, homology, etc.)

- The binding of LEC  [ **CCL16** ]  to  **CCR8**  was much less significant.  Ref: 11470772 Int Immunol, 2001
